# RETRACTED: Gorska-Ponikowska et al. The Major Heat Shock Proteins, Hsp70 and Hsp90, in 2-Methoxyestradiol-Mediated Osteosarcoma Cell Death Model. *Int. J. Mol. Sci.* 2020, *21*, 616

**DOI:** 10.3390/ijms27198512

**Published:** 2026-09-24

**Authors:** Magdalena Gorska-Ponikowska, Alicja Kuban-Jankowska, Antonella Marino Gammazza, Agnieszka Daca, Justyna M. Wierzbicka, Michal A. Zmijewski, Hue H. Luu, Michal Wozniak, Francesco Cappello

**Affiliations:** 1 Department of Medical Chemistry, Medical University of Gdansk, 80-211 Gdansk, Poland; alicjakuban@gumed.edu.pl (A.K.-J.); mwozniak@gumed.edu.pl (M.W.); 2 Euro-Mediterranean Institute of Science and Technology, 90127 Palermo, Italy; antonella.marino@hotmail.it (A.M.G.); francescocappello@iemest.eu (F.C.); 3 Department of Biomedicine, Neurosciences and Advanced Diagnostics (BiND), University of Palermo, 90127 Palermo, Italy; 4 Department of Pathology and Experimental Rheumatology, Medical University of Gdansk, 80-211 Gdansk, Poland; agnieszka.ela@gumed.edu.pl; 5 Department of Histology, Medical University of Gdansk, 80-211 Gdansk, Poland; jwierzbicka@gumed.edu.pl (J.M.W.); mzmijewski@gumed.edu.pl (M.A.Z.); 6 Department of Orthopaedic Surgery and Rehabilitation Medicine, University of Chicago, Chicago, IL 60637, USA; hluu@surgery.bsd.uchicago.edu

The Journal retracts the article “The Major Heat Shock Proteins, Hsp70 and Hsp90, in 2-Methoxyestradiol-Mediated Osteosarcoma Cell Death Model” [1] cited above.

Following publication, concerns were brought to the attention of the Editorial Office regarding the integrity of the figures presented in this article [1].

In accordance with the journal’s standard procedures, the Editorial Office and Editorial Board conducted an investigation that identified duplicated image panels within Figures 4 and 7 of this article [1] and apparent panel duplication between several figures in this article [1] and figures from earlier published materials [2,3,4] produced by a similar group of authors. Although the authors cooperated with the investigation, the Editorial Board deemed the explanations and supporting materials provided insufficient to resolve the identified concerns.

As a consequence, the Editorial Board has lost confidence in the reliability of the reported findings and has decided to retract this publication in accordance with MDPI’s retraction policy.

This retraction was approved by the Editor-in-Chief of the journal *International Journal of Molecular Sciences*.

The authors have been informed of this retraction and have indicated their agreement with this decision. The corresponding author accepts responsibility for the errors and apologizes to the co-authors, the journal, and its readers.
